# Quantification of blood flow in the fetus with cardiovascular magnetic resonance imaging using Doppler ultrasound gating: validation against metric optimized gating

**DOI:** 10.1186/s12968-019-0586-8

**Published:** 2019-11-28

**Authors:** Daniel Ryd, Liqun Sun, Katarina Steding-Ehrenborg, Sebastian Bidhult, Fabian Kording, Christian Ruprecht, Christopher K. Macgowan, Michael Seed, Anthony H. Aletras, Håkan Arheden, Erik Hedström

**Affiliations:** 1 Clinical Physiology, Department of Clinical Sciences Lund, Lund University, Skane University Hospital, Lund, Sweden; 2 Department of Pediatrics, University of Toronto and Hospital for Sick Children, Toronto, ON Canada; 3 Department of Health Sciences, Physiotherapy, Lund University, Lund, Sweden; 4 Department of Biomedical Engineering, Faculty of Engineering, Lund University, Lund, Sweden; 5 Department of Diagnostic and Interventional Radiology, University Medical Center Hamburg-Eppendorf, Hamburg, Germany; 6 Department of Medical Biophysics, University of Toronto and Hospital for Sick Children, Toronto, ON Canada; 7 Department of Diagnostic Imaging, University of Toronto and Hospital for Sick Children, Toronto, ON Canada; 8 School of Medicine, Laboratory of Computing, Medical Informatics and Biomedical, Imaging Technologies, Aristotle University of Thessaloniki, Thessaloniki, Greece; 9 Diagnostic Radiology, Department of Clinical Sciences Lund, Lund University, Skane University Hospital, Lund, Sweden

**Keywords:** Fetal blood flow, Cardiovascular magnetic resonance imaging, Doppler ultrasound gating, Metric optimized gating

## Abstract

**Introduction:**

Fetal cardiovascular magnetic resonance (CMR) imaging is used clinically and for research, but has been previously limited due to lack of direct gating methods. A CMR-compatible Doppler ultrasound (DUS) gating device has resolved this. However, the DUS-gating method is not validated against the current reference method for fetal phase-contrast blood flow measurements, metric optimized gating (MOG). Further, we investigated how different methods for vessel delineation affect flow volumes and observer variability in fetal flow acquisitions.

**Aims:**

To 1) validate DUS gating versus MOG for quantifying fetal blood flow; 2) assess repeatability of DUS gating; 3) assess impact of region of interest (ROI) size on flow volume; and 4) compare time-resolved and static delineations for flow volume and observer variability.

**Methods:**

Phase-contrast CMR was acquired in the fetal descending aorta (DAo) and umbilical vein by DUS gating and MOG in 22 women with singleton pregnancy in gestational week 36^0^ (26^5^–40^0^) with repeated scans in six fetuses. Impact of ROI size on measured flow was assessed for ROI:s 50–150% of the vessel diameter. Four observers from two centers provided time-resolved and static delineations. Bland-Altman analysis was used to determine agreement between both observers and methods.

**Results:**

DAo flow was 726 (348–1130) ml/min and umbilical vein flow 366 (150–782) ml/min by DUS gating. Bias±SD for DUS-gating versus MOG were − 45 ± 122 ml/min (−6 ± 15%) for DAo and 19 ± 136 ml/min (2 ± 24%) for umbilical vein flow. Repeated flow measurements in the same fetus showed similar volumes (median CoV = 11% (DAo) and 23% (umbilical vein)). Region of interest 50–150% of vessel diameter yielded flow 35–120%. Bias±SD for time-resolved versus static DUS-gated flow was 33 ± 39 ml/min (4 ± 6%) for DAo and 11 ± 84 ml/min (2 ± 15%) for umbilical vein flow.

**Conclusions:**

Quantification of blood flow in the fetal DAo and umbilical vein using DUS-gated phase-contrast CMR is feasible and agrees with the current reference method. Repeatability was generally high for CMR fetal blood flow assessment. An ROI similar to the vessel area or slightly larger is recommended. A static ROI is sufficient for fetal flow quantification using currently available CMR sequences.

## Background

Fetal cardiovascular imaging using cardiovascular magnetic resonance (CMR) imaging is increasingly applied for fetal cardiovascular research [1–3] and as a clinical aid for improved diagnosis of congenital cardiovascular malformation [4, 5]. Accurate quantification of fetal blood flow may lead to new insights in fetal cardiovascular physiology. Currently there is no non-invasive ground truth method for measuring fetal blood flow. Furthermore, it has been hypothesized that fetal phase-contrast CMR may improve diagnosis of cardiovascular disease such as coarctation, which is currently underdiagnosed prenatally, by more detailed analysis of aortic flow curve shapes. Phase-contrast CMR provides accurate and precise non-invasive quantification of blood flow in both large and small vessels [6–11], usually gated by electrocardiography [12]. However, in fetal CMR it is currently not possible to obtain a fetal electrocardiogram (ECG) signal of sufficient quality for gating. Post-processing methods are therefore generally used for CMR assessment of fetal cardiovascular physiology. As post-processing is performed offline it is difficult to perform quality control of acquired images in real time, which limits clinical utility. The metric optimized gating (MOG) method is a post-processing method that may currently be considered the non-invasive reference standard for fetal quantitative flow by phase-contrast CMR [13].

Real-time phase-contrast flow, although clinically applicable in adults, lacks sufficient spatial resolution for fetal vessels and is thus not currently available for accurate fetal flow quantification. Direct fetal cardiac gating would enable clinical applicability of fetal quantitative flow measurements by CMR. A recent suggestion to accomplish direct fetal cardiac gating and to overcome time-consuming post-processing is by means of the Doppler ultrasound (DUS) gating method [1, 14]. This method utilizes an MR-compatible ultrasound device to assess blood flow through the beating fetal heart and inputs the Doppler ultrasound waveform into the CMR scanner for gating. It has been validated to ECG-gated phase-contrast quantitative flow measurements in the adult aorta [15] and applied for phase-contrast quantitative flow in the fetal descending aorta [16], but not validated against the current reference method for fetal phase-contrast blood flow measurements.

A potential source of error in flow measurements by phase-contrast CMR is over- or underestimation of flow due to incorrect region of interest (ROI) size or position [9]. To what extent this affects flow volumes and observer variability in fetal flow acquisitions needs to be investigated.

The aims of this study were therefore to 1) validate the DUS against the MOG method for quantifying fetal blood flow in the fetal descending aorta and umbilical vein; 2) assess repeatability of the DUS method; 3) assess the impact of ROI size on fetal flow volume; and 4) compare time-resolved versus static fetal vessel delineations for flow volume and observer variability.

## Methods

### Study population

The Regional Ethical Review Board approved the study, which was performed according to the Helsinki declaration. Pregnant women with singleton pregnancies were recruited at Skane University Hospital, Lund, Sweden. Participants provided written informed consent before participating.

### Gating methods

A prototype CMR-compatible DUS device (Northh Medical GmbH, Hamburg, Germany) was used for external cardiac gating as previously described by Kording et al. [1, 15]. The device assessed flow through the beating fetal heart and converted the Doppler signal into square wave trigger signals transferred to the CMR scanner using an auxiliary coaxial cable. The DUS transducer was placed on the maternal abdomen over the fetal thorax and fastened using an elastic belt. Accurate placement for good signal quality was achieved by testing different positions for the DUS transducer until a consistent Doppler waveform of fetal cardiac blood flow was found. In particular cases, palpation of the fetus was used to simplify the process. The DUS signal can be occasionally lost during imaging due to fetal movement or during maternal deep inhalation or exhalation for breath holds. From previous experience, it often returns spontaneously within approximately 1 min. If the DUS signal was lost and did not return, the position of the transducer was adjusted accordingly in the current study. A consistent Doppler waveform of fetal cardiac blood flow was verified before acquisition of flow data.

For the MOG method, flow data were acquired using a simulated heart rate with a constant RR interval of 525 ms in order to oversample the fetal cardiac cycle as proposed in the original description of MOG for fetal CMR [13]. Image reconstruction was performed offline using the MOG-Public Software 2.7 (https://github.com/MetricOptimizedGating/MOG-Public). In short, the MOG algorithm was applied to the oversampled phase-contrast raw data, taking approximately 10 min per slice. Data were then transferred back to the CMR scanner for reconstruction of standard DICOM images for analysis. The current study used the initial MOG algorithm that assumes a constant fetal heart rate for the first and second halves of data acquisition. Although a more recent MOG implementation accounting for beat-to-beat variation now exists, it requires time-consuming post-processing and is in our experience less important for flow measurements than cardiac cine imaging (unpublished data).

### Image acquisition

CMR was performed using a 1.5 T scanner (Aera, Siemens, Erlangen, Germany) with a combination of an 18-channel phased-array coil and a spine receiver coil. Data were acquired in the left decubitus position to avoid compression of the maternal inferior caval vein and potential impact on cardiovascular physiology during CMR. All CMR acquisitions were performed within a maximum limit for specific absorption rate of 2 W/kg and with limited noise levels.

**Fig. 1:**
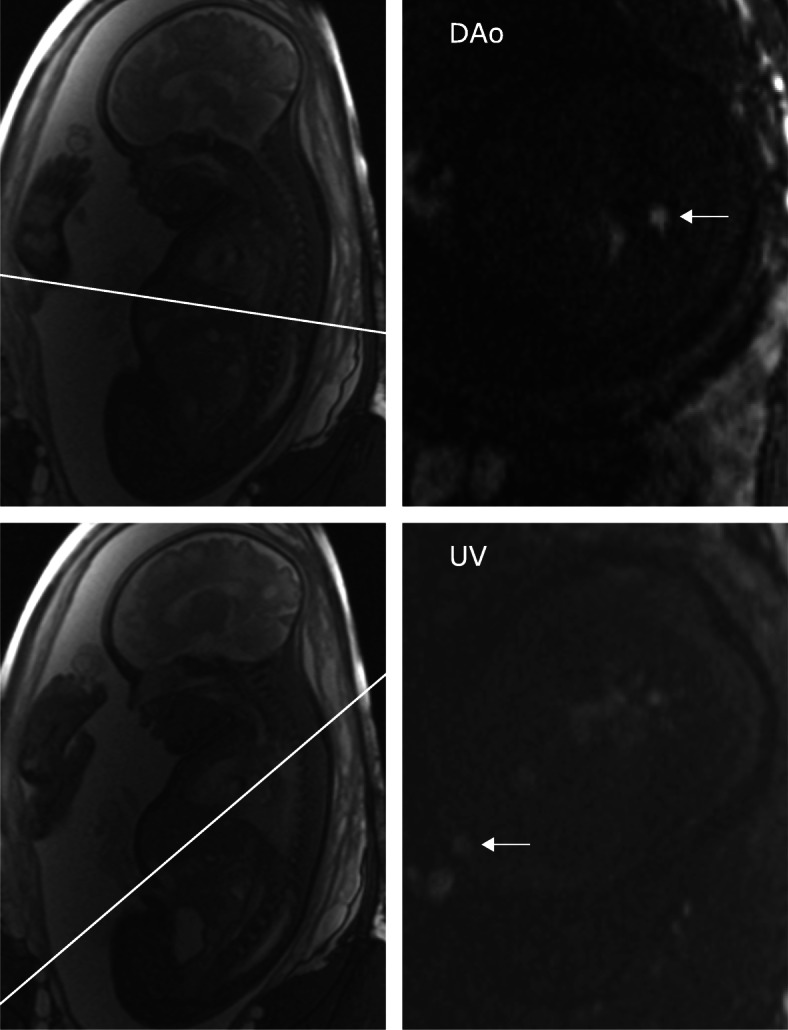
Flow acquisitions in the fetal descending aorta (DAo) and umbilical vein (UV). Balanced steady-state free precession localizers (left) were used to plan phase-contrast acquisitions (right). Localizers were acquired in several planes to confirm that flow acquisitions were perpendicular to the vessel of interest. White lines indicate the descending aorta (top) and umbilical vein (bottom) scan planes and arrows indicate the respective vessel of interest

Sagittal, coronal and transversal balanced steady-state free precession (bSSFP) images were acquired to localize the fetal descending aorta and the umbilical vein. Additional scout images were acquired if necessary to certify that the phase-contrast flow acquisitions were positioned perpendicular to the respective vessel. Phase-contrast quantitative flow data were acquired in the fetal descending aorta at the level of the diaphragm and in the fetal intra-abdominal umbilical vein (Fig. 1). In six fetuses, DUS-gated flow was acquired six to 12 times in both the fetal descending aorta and the umbilical vein, respectively, without repositioning the pregnant woman, to assess repeatability of DUS-gated flow measurements. Three of these six fetuses were included only to test the repeatability of the DUS-gated flow and no corresponding MOG flow images were acquired. A 2D segmented gradient recalled echo phase-contrast sequence was used during shallow maternal free breathing to limit acquisition time and reduce the likelihood of fetal movement during image acquisition. Parameters were the same for all subjects, with the exception of DUS acquisition time (TE/TR 2.76/5.03 ms, flip angle 20°, VENC 150 cm/s, in-plane resolution 1.41 × 1.41 mm, slice thickness 5 mm, acquired temporal resolution 30.18 ms, reconstructed temporal resolution 14.7 ms). Acquisition time for DUS was median 22 s (range 19–31 s) and for MOG 26 s in all acquisitions.

### Image analysis

Images acquired by DUS gating and images reconstructed using MOG were analyzed in Segment v2.2 (Medviso AB, Lund, Sweden) [17]. Vessel delineations were performed using the same static ROI throughout the cardiac cycle or using a time-resolved ROI adjusted to the vessel contour in each time frame. Static ROI:s of different sizes were used to determine the impact of ROI size on measured flow. A circular ROI fitted to the vessel contour in the time frame with the largest vessel area was used as reference. Additional circular ROI:s were placed at the same ROI center with diameters 50, 75, 125 and 150% of that of the original 100% ROI. Flow volumes were obtained for each ROI size and expressed as percentage of the flow volume of the original 100% ROI.

Four observers at two centers (observer 1, DS, and 2, EH, in Lund; observer 3, CKM, and 4, LS, in Toronto) manually delineated the fetal descending aorta and the umbilical vein using both time-resolved and static delineations. Data are presented based on delineations by observer 1, if not otherwise stated. For time-resolved delineations an ROI was drawn around the vessel lumen in all time frames of the cardiac cycle and adapted to vessel diameter over time. For static delineations, the largest vessel area was used to determine the static ROI size and the ROI copied to all time frames of the cardiac cycle. All observers were blinded to each others’ delineations and to subject information. Observer 1 delineated all vessels twice to assess intraobserver variability. Flow assessment was generally performed without background correction. Agreement of measurements without and with linear background correction was assessed for time-resolved measurements and the impact of ROI size on flow measurements was assessed both without and with linear background correction.

### Statistical analysis

All statistical analyses were performed using GraphPad (Prism v8.0.2, La Jolla, California, USA). Data in text are presented as mean ± standard deviation (SD) or median (range) as appropriate. Bias and variability were assessed as the mean difference of measurements ± SD. Coefficient of variation (CoV) was calculated as SD divided by the mean of differences and expressed as percentage. Bland-Altman analysis was performed to determine bias and 95% limits of agreement (LoA, i.e. bias ±1.96SD, which is presented in Bland-Altman graphs) for flow without and with linear background correction, time-resolved versus static delineations, intra- and interobserver agreement and agreement between DUS and MOG methods.

## Results

**Fig. 2:**
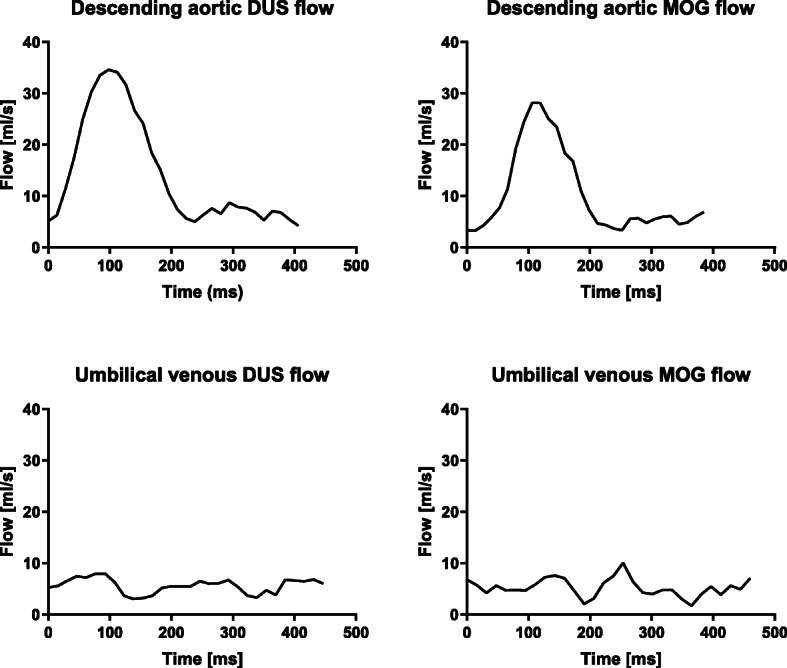
Typical flow curves for the fetal descending aorta and umbilical vein by Doppler ultrasound (DUS) gating and metric optimized gating (MOG). An example of flow curves for the fetal descending aorta (top) and umbilical vein (bottom) from one fetus obtained using the DUS (left) and MOG (right) methods. Note the difference between methods in descending aorta peak flow and umbilical vein flow variability over time

This study included 22 women of median age 31 (25–43) years with singleton pregnancy in gestational week 36^0^ (26^5^–40^0^) (Table 1). Three of these were included for DUS repeatability only. The remaining 19 fetuses had flow images acquired using both the DUS and MOG methods. Among these 19 fetuses, the number of successfully acquired flow images in the fetal descending aorta were *n* = 19 for both DUS and MOG, and in the umbilical vein *n* = 15 using DUS and n = 15 using MOG (of which *n* = 14 had both DUS and MOG). The main reason for exclusion of the *n* = 5 fetuses lacking overlapping data for the umbilical vein was low image quality, speculatively related to fetal movements. For the repeatability study, data acquisition was not possible in fetus 6 for umbilical vein, likely due to major fetal movement combined with a small fetus. Typical flow curves obtained in the descending aorta and umbilical vein using the DUS and MOG methods are shown in Fig. 2. This figure illustrates the strength of the DUS method for accurately measuring aortic peak flow.

**Table 1:** Population characteristics

Fetal heart rate by DUS (bpm)	136 (120–152)
Gestational age (weeks)	36^0^ (26^5^–40^0^)
Maternal body mass index (kg/m^2^)	30 (19–37)
Maternal height (cm)	165 (157–173)
Maternal weight (kg)	79 (49–109)
Maternal age (years)	31 (25–43)

*DUS* Doppler ultrasound

### DUS versus MOG flow measurements

**Fig. 3:**
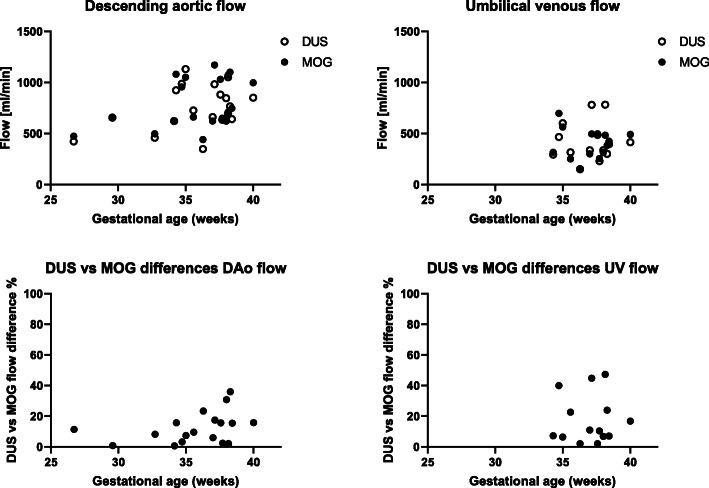
Flow volumes and flow differences between methods versus gestational age. The graphs show flow volumes obtained using time-resolved delineations without background correction versus gestational age for the Doppler ultrasond (DUS) and metric optimized gating (MOG) methods in the descending aorta (top left) and umbilical vein (top right) and the percent difference in flow between the DUS and MOG methods versus gestational age for the descending aorta (DAo; bottom left) and umbilical vein (UV; bottom right)

Descending aortic flow was 726 (348–1130) ml/min by DUS and 708 (440–1170) ml/min by MOG and umbilical venous flow was 366 (150–782) ml/min by DUS and 404 (147–697) ml/min by MOG. Figure 3 shows flow volumes by DUS and MOG and relative flow differences between methods versus gestational age. Bias±SD for flow measurements without versus with linear background correction was for descending aortic flow − 17 ± 42 ml/min (− 3 ± 6%) by DUS and − 17 ± 43 ml/min (− 3 ± 6%) by MOG, respectively, and for umbilical venous flow 33 ± 41 ml/min (8 ± 12%) by DUS and 43 ± 42 ml/min (14 ± 17%) by MOG, respectively.

**Fig. 4:**
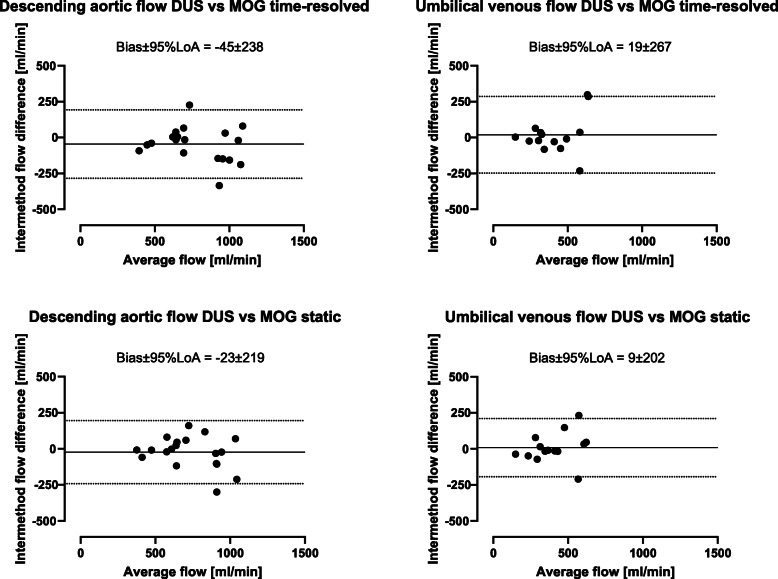
Doppler-ultrasound (DUS) versus metric optimized gating (MOG) flow volumes. The DUS versus MOG comparison showed low bias and wide limits of agreement between methods. Bland-Altman plots showing intermethod agreement for flow volume quantification in the descending aorta (left) and umbilical vein ( right) using time-resolved (top) and static (bottom) delineations, respectively. Solid lines indicate bias and dotted lines indicate 95% limits of agreement (LoA)

Flow volumes obtained using the DUS and MOG methods showed a low bias, although with wide limits of agreement. For time-resolved delineations, bias and variability between the DUS and MOG methods for descending aortic flow and umbilical venous flow were − 45 ± 122 ml/min (− 6 ± 15%) and 19 ± 136 ml/min (2 ± 24%) (Fig. 4). For static delineations, bias and variability between the DUS and MOG methods for descending aortic flow and umbilical venous flow were − 23 ± 112 ml/min (− 2 ± 14%) and 9 ± 103 ml/min (0 ± 23%) (Fig. 4). Aliasing was present in one fetus in aortic flow data after but not before MOG reconstruction, thus not visible at acquisition. This aliased flow data set could not be unwrapped. In the same fetus aliasing was present also in the DUS-gated aortic flow data, but this flow data set could be successfully unwrapped.

### Repeated flow acquisitions using the DUS method

**Fig. 5:**
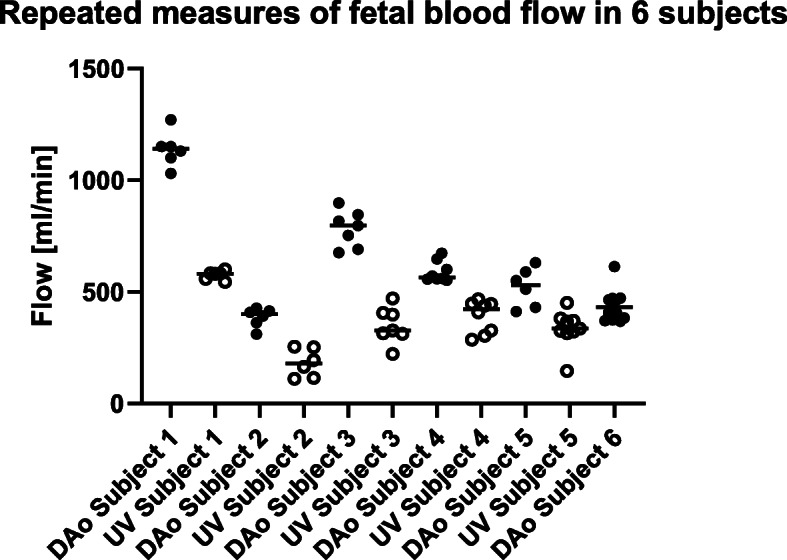
Repeated flow acquisitions in six fetuses. The Doppler ultrasound gating method showed generally high repeatability. Measurements from repeated flow acquisitions in the descending aorta (closed circles) and umbilical vein (open circles) in six subjects. Flow acquisitions were acquired six to 12 times in each vessel. In subject 6 umbilical venous flow was not successfully acquired due to early termination of the CMR examination. Solid lines indicate medians

Flow data from fetuses in whom DUS-gated phase-contrast images were acquired repeatedly in the fetal descending aorta and abdominal umbilical vein are shown in Fig. 5 and in Table 2. Repeated flow acquisitions by DUS gating in the same fetus showed similar flow volumes. In one subject (subject 2) one repeated umbilical venous flow was acquired at a registered fetal heart rate of 80 beats per minute. This value is far below normal and most likely represents a poor DUS signal with missed heart beat detection rather than the true fetal heart rate.

**Table 2:** Flow volumes (ml/min) from repeated measurements in six subjects. Data are presented as median [interquartile range] (CoV)

Subject	Descending aortic flow	Umbilical venous flow
Subject 1	1140 [97] (CoV = 7%)	581 [33] (CoV = 3%)
Subject 2	400 [68] (CoV = 11%)	179 [140] (CoV = 35%)
Subject 3	797 [155] (CoV = 10%)	327 [93] (CoV = 23%)
Subject 4	565 [78] (CoV = 8%)	423 [138] (CoV = 19%)
Subject 5	531 [174] (CoV = 17%)	335 [58] (CoV = 25%)
Subject 6	432 [93] (CoV = 16%)	Unsuccessful image acquisition

### Impact of region of interest size on flow measurements

**Fig. 6:**
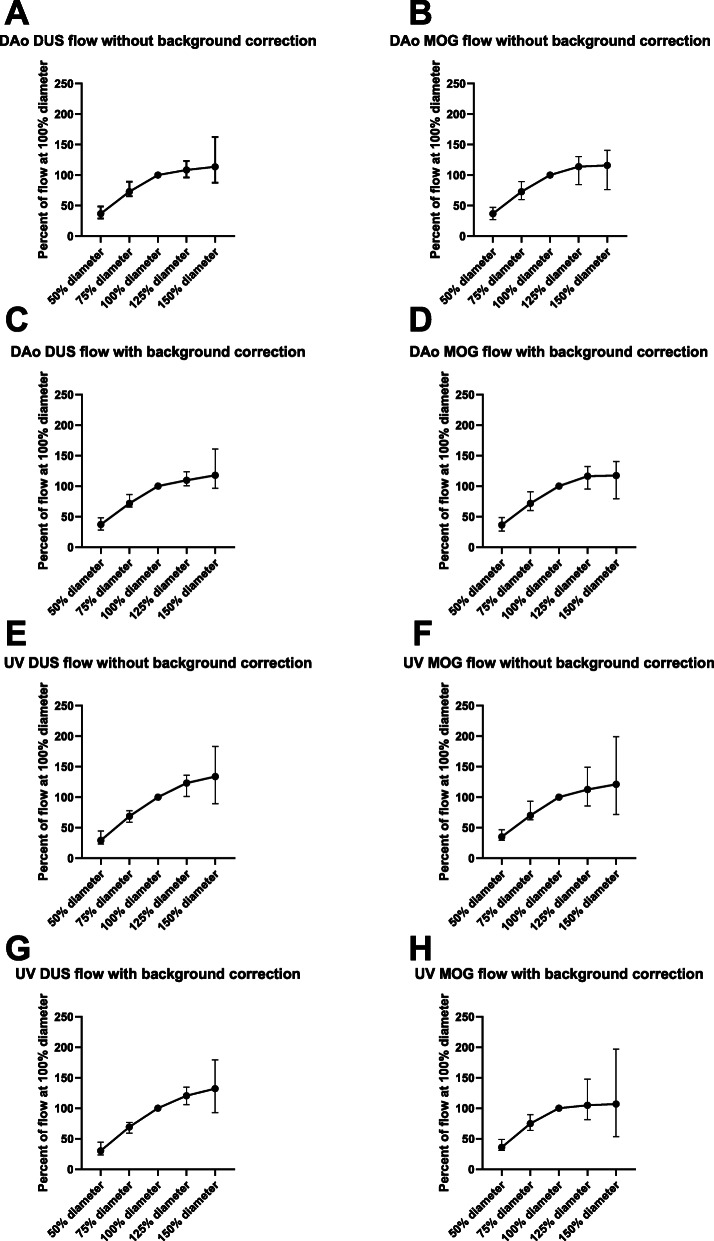
Impact of region of interest (ROI) size on measured flow volumes. The underestimation of flow caused by an ROI smaller than actual vessel size is relatively greater than the overestimation of flow caused by an ROI larger than the vessel. Thus, the ROI should contain the vessel or be slightly larger, but not smaller, than actual vessel size. Flow volumes for ROI sizes 50, 75, 125 and 150% diameter were expressed as percent of flow volume of the 100% vessel diameter. Data are shown for the descending aorta (DAo; **a**-**d**) and umbilical vein (UV; **e**-**h**) using the Doppler-ultrasound gating (DUS; left) and metric optimized gating (MOG; right) methods both without and with linear background correction. Median and range error bars are indicated

Data from flow measurements performed using varying ROI sizes are shown in Fig. 6. For flow measurements both without and with linear background correction, increase in ROI size to greater than vessel diameter caused flow volume overestimation and decrease in ROI size to smaller than vessel diameter caused flow volume underestimation. The underestimation was however relatively greater than the overestimation caused by increased ROI size.

### Time-resolved versus static delineations

**Fig. 7:**
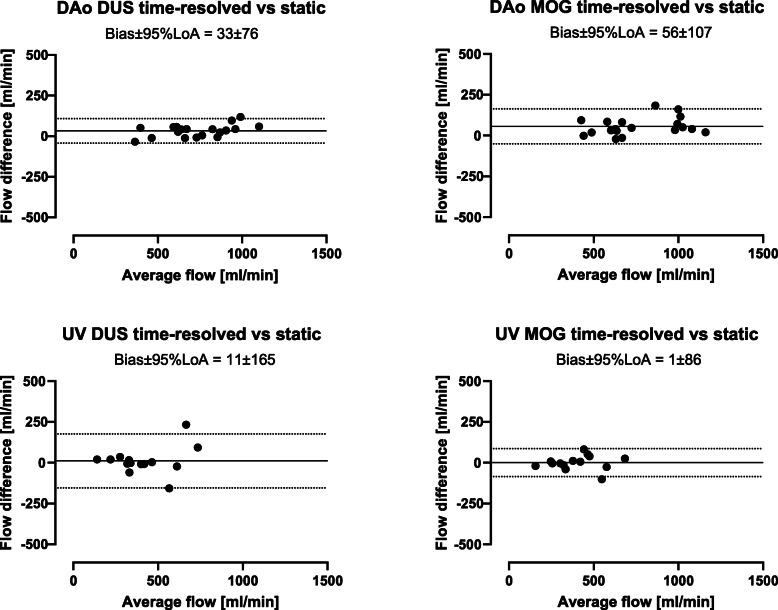
Time-resolved versus static delineations. The variability between time-resolved and static delineations is small and static delineations can thus currently be recommended for fetal blood flow measurements in the descending aorta and umbilical vein. Bland-Altman plots showing flow volumes obtained using time-resolved versus static vessel delineations of the fetal descending aorta (DAo; top) and umbilical vein (UV; bottom) using the Doppler ultrasound (DUS; left) and metric optimized gating (MOG; right) methods. Solid lines indicate bias and dotted lines indicate 95% limits of agreement (LoA)

Time-resolved versus static vessel delineations showed low bias and narrow limits of agreement (Fig. 7). Time-resolved versus static delineations showed a bias and variability for descending aortic flow of 33 ± 39 ml/min (4 ± 6%) using the DUS method and 56 ± 55 ml/min (7 ± 7%) using the MOG method, and for umbilical venous flow 11 ± 84 ml/min (2 ± 15%) using the DUS method and 1 ± 44 ml/min (0 ± 10%) using the MOG method.

### Intra- and interobserver variability

**Fig. 8:**
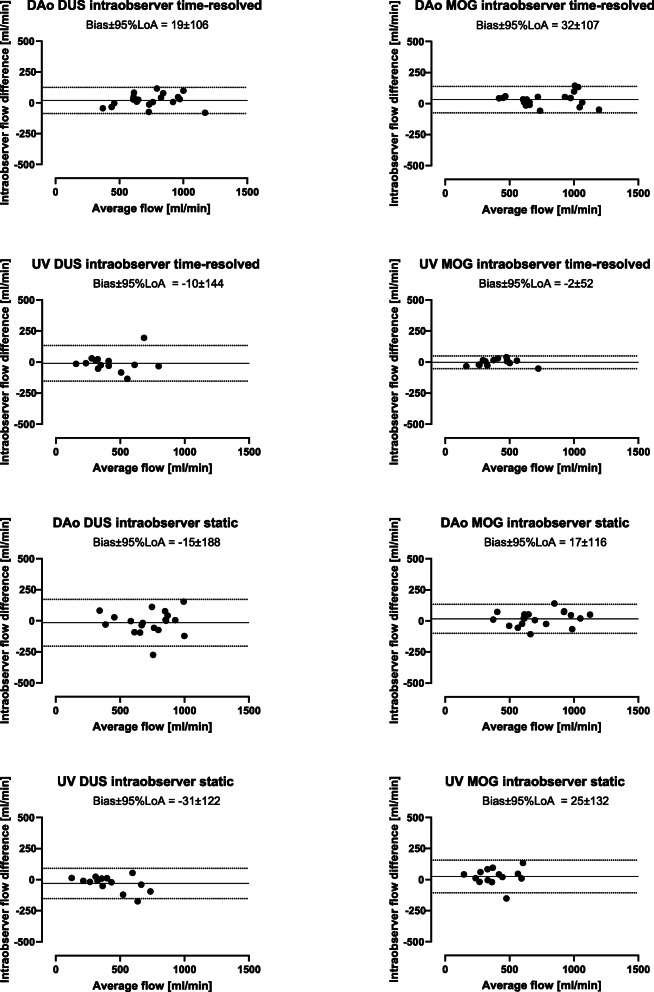
Intraobserver measurements by time-resolved and static delineations. Intraobserver variability was low for both time-resolved and static delineations, using both the DUS and MOG methods. Bland-Altman plots showing intraobserver agreement using time-resolved and static delineations of the fetal descending aorta (DAo) and umbilical vein (UV) using the Doppler ultrasound (DUS; left) and metric optimized gating (MOG; right) methods. Solid lines indicate bias and dotted lines indicate 95% limits of agreement (LoA)

For time-resolved delineations, intraobserver variability for DUS and MOG methods were for descending aortic flow 19 ± 54 ml/min (2 ± 7%) and 32 ± 55 ml/min (4 ± 6%), respectively, and for umbilical venous flow − 10 ± 73 ml/min (− 3 ± 13%) and − 2 ± 26 ml/min (− 1 ± 8%), respectively (Fig. 8). For static delineations, intraobserver variability for DUS and MOG methods were for descending aortic flow − 15 ± 96 ml/min (− 2 ± 13%) and 17 ± 59 ml/min (2 ± 9%), respectively, and for umbilical venous flow − 31 ± 62 ml/min (− 5 ± 11%) and 25 ± 67 ml/min (8 ± 17%), respectively (Fig. 8).

**Fig. 9:**
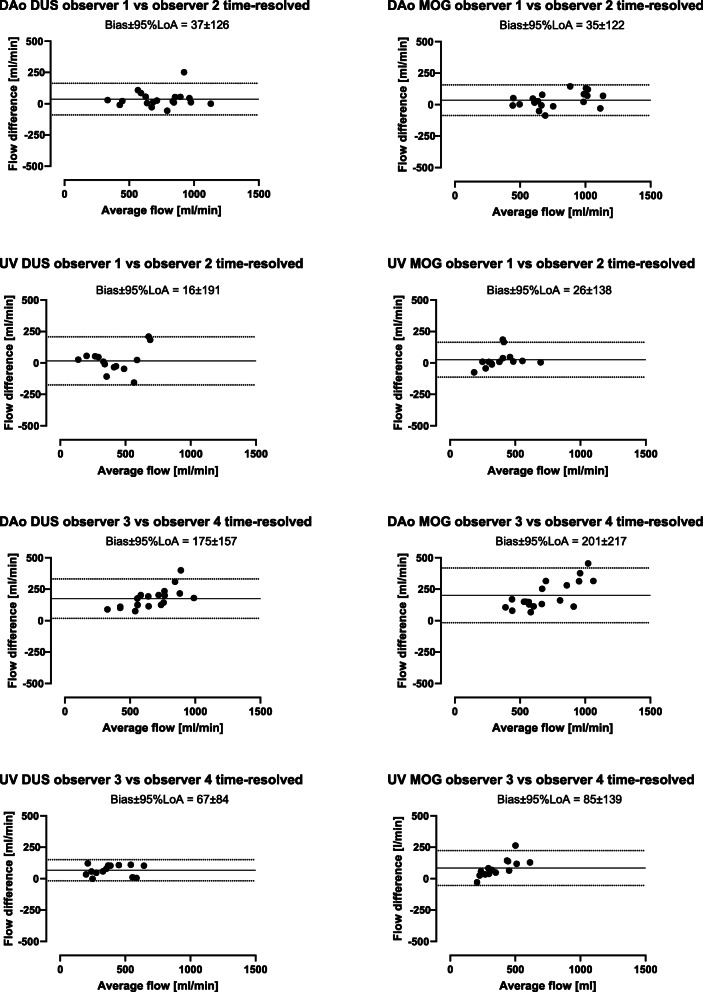
Intracenter interobserver measurements by time-resolved delineations. Interobserver variability was low in one center but showed high bias in the other center, suggesting that consensus for how to delineate fetal vessels is needed. Bland-Altman plots showing interobserver measurements between observer 1 and 2 (same center) and 3 and 4 (same center) using time-resolved delineations of the fetal descending aorta (DAo) and umbilical vein (UV) using the Doppler-ultrasound (DUS; left) and metric optimized gating (MOG; right) methods. Solid lines indicate bias and dotted lines indicate 95% limits of agreement (LoA)

Interobserver variability was low between three of four observers. Interobserver variability was low in center one and between center one and one observer from center two whereas the second observer from center two measured systematically lower flow volumes. Interobserver variability between all observers is reported in Table 3 (time-resolved delineations) and Table 4 (static delineations). For time-resolved delineations, interobserver variability within centers is shown in Fig. 9.

**Table 3:** Interobserver variability for flow measurements using time-resolved delineations. Data are presented as mean interobserver differences ± SD in ml/min and percent of average flow

Vessel	Observer 1 vs 2	Observer 1 vs 3	Observer 1 vs 4	Observer 3 vs 4
Descending aorta DUS	37 ± 65 (5 ± 8%)	0 ± 64 (0 ± 9%)	175 ± 87 (26 ± 9%)	175 ± 80 (26 ± 8%)
Descending aorta MOG	35 ± 62 (4 ± 8%)	−3 ± 80 (0 ± 9%)	198 ± 99 (28 ± 10%)	201 ± 111 (28 ± 10%)
Umbilical vein DUS	16 ± 98 (4 ± 20%)	2 ± 84 (−2 ± 23%)	69 ± 71 (17 ± 14%)	67 ± 43 (19 ± 14%)
Umbilical vein MOG	26 ± 71 (4 ± 21%)	−9 ± 48 (−3 ± 13%)	75 ± 61 (18 ± 19%)	85 ± 71 (20 ± 15%)

*DUS* Doppler ultrasound, *MOG* Metric Optimized Gating

**Table 4:** Interobserver variability for flow measurements using static delineations. Data are presented as mean interobserver differences ± SD in ml/min and percent of average flow

Vessel	Observer 1 vs 2	Observer 1 vs 3	Observer 1 vs 4	Observer 3 vs 4
Descending aorta DUS	−17 ± 66 (−2 ± 11%)	−43 ± 55 (−5 ± 7%)	98 ± 107 (15 ± 17%)	142 ± 125 (21 ± 17%)
Descending aorta MOG	−13 ± 64 (−2 ± 8%)	−85 ± 88 (−10 ± 9%)	63 ± 114 (9 ± 14%)	148 ± 152 (20 ± 16%)
Umbilical vein DUS	4 ± 66 (2 ± 16%)	−30 ± 69 (−9 ± 23%)	24 ± 83 (6 ± 19%)	53 ± 61 (15 ± 20%)
Umbilical vein MOG	17 ± 86 (2 ± 22%)	7 ± 88 (2 ± 21%)	45 ± 88 (13 ± 24%)	38 ± 64 (11 ± 19%)

*DUS* Doppler ultrasound, *MOG* Metric Optimized Gating

### Relative efficiency of techniques

The number of repeated scans required for flow images in subjects where both DUS and MOG acquisitions were successful were for the descending aorta 1 (1–3) by DUS and 1 (1–3) by MOG and for the umbilical vein 1 (1–3) by DUS and 1 (1–2) by MOG. The scan time is similar for both DUS and MOG, as the same sequences are used for image acquisition. The DUS device typically takes less than 5 min to position and may in some cases need repositioning as described in Methods/Gating methods. For each MOG flow image, applying the MOG algorithm offline took approximately 10 min (i.e. 20 min per fetus if only one acquisition for each vessel was performed). After applying the offline MOG algorithm, data need to be transferred back to the scanner for image reconstruction. In our hospital the scanner is available for this after regular working hours. In total, reconstructed MOG flow images for analysis thus take considerably longer to acquire. Moreover, a static vessel delineation typically takes about 20 s to perform. A time-resolved vessel delineation on the other hand consists of about 30 static delineations and takes about 5 min to perform.

## Discussion

This study provides insights into assessment of fetal blood flow by CMR. Quantification of blood flow in the fetal descending aorta and umbilical vein using DUS-gated phase-contrast CMR is feasible and agrees with the current reference method metric optimized gating. Repeatability was generally high for fetal blood flow assessment by DUS-gated CMR. Underestimation of vessel size caused an underestimation of measured flow volumes, which was relatively greater than the overestimation of measured flow caused by overestimation of vessel size. An ROI similar to the vessel area or slightly larger, but not smaller, is thus recommended. Time-resolved versus static delineations showed low variability. A static ROI is thus sufficient for fetal flow quantification using currently available CMR sequences. Interobserver variability was low between three of four observers and inter-center consensus for delineations are crucial for future studies on fetal cardiovascular physiology utilizing fetal phase-contrast CMR.

There are several methodological differences between the DUS and MOG methods for flow data acquisition, which may explain the observed variability in measured flow volumes. Gating by DUS is similar to conventional ECG gating in that cardiac activity is registered continuously throughout image acquisition. As the DUS method registers beat-to-beat variation it is potentially accurate even with considerable fetal heart rate variation during image acquisition. Metric optimized gating utilizes numerical optimization instead of direct cardiac gating and is based on the principle that data are acquired during a time period greater than the RR interval so that oversampled data is used for reconstruction [13]. Image reconstruction is performed retrospectively by synchronizing data for a large number of hypothetical RR-intervals and the reconstruction with minimal misgating artifacts is chosen. The available MOG software for the CMR scanner used in the current study on the other hand used a two-parameter model of the fetal heart rate which assumes a fixed fetal heart rate during the first and second halves of the image acquisition respectively, whereas in reality the fetal heart rate may vary considerably [18]. Thus, in fetuses with a large heart rate variation during image acquisition this model may be inaccurate [13]. More recent implementations of MOG account for beat-to-beat changes in heart rate to alleviate this limitation [19–21].

Another possible explanation for the differences in flow volumes obtained by the DUS and MOG methods in this study is that the DUS gating signal could not be recorded simultaneously with the MOG data acquisition, as the scanner used in the current study allows only one channel for ECG signals. Thus the DUS signal and simulated ECG as needed for MOG could not be saved simultaneously. Instead, DUS-gated image acquisitions were performed immediately after the MOG image acquisitions. Flow volumes obtained by the DUS method could thus differ from those obtained by MOG due to physiological changes in flow over time or due to fetal movement between acquisitions. Although suspected fetal movement was investigated and adapted for by acquiring new scout images, small changes in fetal position could have been missed. It was noted in one fetus that aliasing was present in the aortic flow after but not before MOG reconstruction. This aliased flow data set could not be unwrapped. Velocity aliasing distorts the flow data and may lead to MOG misgating and underestimation of the flow curve, making aliased flow data difficult to unwrap. In the same fetus, aliasing was present also in the DUS-gated aortic flow. However, the DUS-gated aliased aortic flow was successfully unwrapped. This suggests that the MOG method may be limited by the inability to detect aliasing during CMR examination and to unwrap aliased flow data.

In the six fetuses in whom descending aortic and umbilical venous flow images were acquired repeatedly, flow volumes obtained in the same fetus were generally similar. The observed variability can be explained by both measurement error and physiological variability in fetal blood flow, the latter also more likely to potentially confound the DUS versus MOG comparison in the current study. Relatively large short-term variability in descending aortic flow has been shown in the fetal lamb, almost always occurring simultaneously with fetal respiratory movements [22]. Rapid irregular fetal respiratory movements occurs with increasing heart rate and descending aortic flow whereas other patterns of respiratory movements occasionally occurs with decreasing heart rate and descending aortic flow [22]. Umbilical venous flow, however, has been shown to decrease slightly in conjunction with rapid irregular fetal respiratory movements and to decrease markedly in conjunction with other patterns of fetal respiratory movements in lamb [23]. Thus, the observed variability in individual fetal descending aortic and umbilical venous flow volumes in the current study could be related to fetal respiratory movement during image acquisitions. Other potential mechanisms causing variation in fetal blood flow include fetal movement and uterine contractions, however not further assessed in the current study.

Region of interest size versus vessel size affects measured flow volumes both when the ROI is larger and smaller than the actual vessel size. Region of interest diameters of 125 and 150% of that of the actual vessel diameter overestimated flow volumes by 15 and 20%, respectively. The current results are similar to those in a previous study on the accuracy of phase-contrast CMR for quantification of flow in a small vessel phantom with simulated cardiac motion [9]. In that study, Arheden et al. showed that as ROI size increased the peak increase in measured flow was approximately 20% of true flow volume at an ROI area approximately twice the true vessel area [9]. In contrast, measured flow volumes rapidly declined as ROI size decreased below the actual vessel size [9], as also confirmed in the current study in human fetuses. It should be noted that with the current CMR protocols, measurements of fetal flow are at the limit of required spatial resolution and that partial volume effects are likely to impact measured flow [24], which may at least in part explain the increase in measured flow with an ROI larger than the vessel. Measurement errors must be minimized to allow detailed study of fetal cardiovascular physiology, and erroneous delineation of vessels will also lead to misdiagnosis in clinical applications. Thus the ROI is recommended to cover the vessel area or be slightly larger, but not smaller than the vessel.

The time-resolved versus static delineations showed a small bias for both the DUS and MOG methods for both descending aortic flow and umbilical venous flow. However, the results of the current study show that static delineations provide similar flow volumes. Thus, considering the current limitations of spatial resolution in fetal phase-contrast CMR, static delineations may currently be recommended as they result in similar flow volumes, are less time-consuming to perform and depending on training and consensus between centers may decrease variability between observers. With increased spatial resolution of newer CMR sequences or assessment at 3 Tesla and when acquisition of vessel area change over the cardiac cycle is of interest, as for assessment of vessel compliance, time-resolved delineations should however be advocated. Estimations of blood flow in the human fetal descending aorta and umbilical vein have previously been performed using ultrasonography and CMR [11, 13, 25–28]. Flow volumes obtained in the current study are comparable to these results.

The time-consuming post-processing required for MOG reconstruction can be avoided if the DUS method is used. It was noted in the current study that both technologists and junior doctors with no previous experience of fetal imaging and little or no experience in obstetrics correctly placed the DUS transducer after only short training. Thus, DUS-gated phase-contrast quantitative flow is suitable for clinical routine application. However, success rate may be lower in smaller fetuses, fetuses with complex cardiac malformation or obese pregnant women. Development of a multi-probe DUS device may in part overcome challenges in acquiring a consistent gating signal.

### Limitations

This study included mainly third trimester fetuses. The current results may therefore not apply to smaller fetuses in whom fetal movement may have larger impact on stability of DUS signal and image acquisition. In addition, flow measurements in smaller fetuses may be unreliable as current standard methods are at their limit of spatial resolution versus vessel size. Development of high-resolution CMR sequences for fetal application however shows promising results. Moreover, all images were acquired in maternal free breathing and the possible effects of maternal breathing movement on quantification of fetal flow volumes was not investigated. However, as both DUS and MOG data were acquired under similar conditions, it is unlikely that the shallow maternal breathing had impact on the comparison in the current study. Finally, a direct comparison of trigger signals between the DUS and MOG methods was not possible as the DUS gating signal could not be recorded simultaneously with the MOG data acquisition and the post-processed MOG gating signal was not available for further analysis offline.

## Conclusions

This study provides insights into assessment of fetal blood flow by CMR. Quantification of blood flow in the fetal descending aorta and umbilical vein using DUS-gated phase-contrast CMR is feasible and agrees with the current reference method metric optimized gating. Repeatability was generally high for fetal blood flow assessment. An ROI similar to the vessel area or slightly larger, but not smaller, is recommended and a static ROI is sufficient for fetal flow quantification using currently available CMR sequences and may decrease variability between studies. Interobserver variability was low between three of four observers and inter-center consensus for delineations are crucial for future studies on fetal cardiovascular physiology utilizing fetal phase-contrast CMR. The DUS method can be used with little training and requires no post-processing, making it easily applied clinically. In cases where a DUS signal however cannot be obtained the MOG method may still provide data for flow measurements.

## Data Availability

The datasets used during the current study are available from the corresponding author on reasonable request.

## Abbreviations

bSSFP: Balanced steady state free precession
CMR: Cardiovascular magnetic resonance
DAo: Descending aorta
ECG: Electrocardiogram
DUS: Doppler ultrasound
MOG: Metric optomized gaing
ROI: Region of interest
UV: Umbilical vein
