# Correction to: Quantification of blood flow in the fetus with cardiovascular magnetic resonance imaging using Doppler ultrasound gating: validation against metric optimized gating

**DOI:** 10.1186/s12968-022-00850-8

**Published:** 2022-04-01

**Authors:** Daniel Ryd, Liqun Sun, Katarina Steding-Ehrenborg, Sebastian Bidhult, Fabian Kording, Christian Ruprecht, Christopher K. Macgowan, Michael Seed, Anthony H. Aletras, Håkan Arheden, Erik Hedström

**Affiliations:** 1 Clinical Physiology, Department of Clinical Sciences Lund, Lund University, Skane University Hospital, Lund, Sweden; 2 Department of Pediatrics, University of Toronto and Hospital for Sick Children, Toronto, ON Canada; 3 Department of Health Sciences, Physiotherapy, Lund University, Lund, Sweden; 4 Department of Biomedical Engineering, Faculty of Engineering, Lund University, Lund, Sweden; 5 Department of Diagnostic and Interventional Radiology, University Medical Center Hamburg-Eppendorf, Hamburg, Germany; 6 Department of Medical Biophysics, University of Toronto and Hospital for Sick Children, Toronto, ON Canada; 7 Department of Diagnostic Imaging, University of Toronto and Hospital for Sick Children, Toronto, ON Canada; 8 School of Medicine, Laboratory of Computing, Medical Informatics and Biomedical, Imaging Technologies, Aristotle University of Thessaloniki, Thessaloniki, Greece; 9 Diagnostic Radiology, Department of Clinical Sciences Lund, Lund University, Skane University Hospital, Lund, Sweden

## Correction to: Journal of Cardiovascular Magnetic Resonance (2019) 21:74 https://doi.org/10.1186/s12968-019–0586-8

Following publication of the original article [1] the author name ‘Daniel Salehi’ has been updated to ‘Daniel Ryd’.
